# Cellular processing of α-synuclein fibrils results in distinct physiological C-terminal truncations with a major cleavage site at residue Glu 114

**DOI:** 10.1016/j.jbc.2023.104912

**Published:** 2023-06-10

**Authors:** Stephan Quintin, Grace M. Lloyd, Giavanna Paterno, Yuxing Xia, Zachary Sorrentino, Brach M. Bell, Kimberly-Marie Gorion, Edward B. Lee, Stefan Prokop, Benoit I. Giasson

**Affiliations:** 1 Department of Neuroscience, College of Medicine, University of Florida, Gainesville, Florida, USA; 2 Center for Translational Research in Neurodegenerative Disease, College of Medicine, University of Florida, Gainesville, Florida, USA; 3 Department of Neurosurgery, College of Medicine, University of Florida, Gainesville, Florida, USA; 4 Department of Pathology and Laboratory Medicine, University of Pennsylvania (PENN) School of Medicine, Philadelphia, Pennsylvania, USA; 5 Department of Pathology, College of Medicine, University of Florida, Gainesville, Florida, USA; 6 McKnight Brain Institute, College of Medicine, University of Florida, Gainesville, Florida, USA

**Keywords:** carboxy-terminal, cleavage, neurodegeneration, pathology, prion, α-synuclein

## Abstract

α-synuclein (αS) is an abundant, neuronal protein that assembles into fibrillar pathological inclusions in a spectrum of neurodegenerative diseases that include Lewy body diseases (LBD) and Multiple System Atrophy (MSA). The cellular and regional distributions of pathological inclusions vary widely between different synucleinopathies contributing to the spectrum of clinical presentations. Extensive cleavage within the carboxy (C)-terminal region of αS is associated with inclusion formation, although the events leading to these modifications and the implications for pathobiology are of ongoing study. αS preformed fibrils can induce prion-like spread of αS pathology in both *in vitro* and animal models of disease. Using C truncation-specific antibodies, we demonstrated here that prion-like cellular uptake and processing of αS preformed fibrils resulted in two major cleavages at residues 103 and 114. A third cleavage product (122 αS) accumulated upon application of lysosomal protease inhibitors. *In vitro*, both 1-103 and 1-114 αS polymerized rapidly and extensively in isolation and in the presence of full-length αS. 1-103 αS also demonstrated more extensive aggregation when expressed in cultured cells. Furthermore, we used novel antibodies to αS cleaved at residue Glu114, to assess x-114 αS pathology in postmortem brain tissue from patients with LBD and MSA, as well as three different transgenic αS mouse models of prion-like induction. The distribution of x-114 αS pathology was distinct from that of overall αS pathology. These studies reveal the cellular formation and behavior of αS C-truncated at residues 114 and 103 as well as the disease dependent distribution of x-114 αS pathology.

Synucleinopathies are a spectrum of neurodegenerative diseases pathologically characterized by the presence of central nervous system (CNS) α-synuclein (αS) inclusions (1, 2, 3). In some of these disorders, such as Parkinson’s disease (PD) and dementia with Lewy bodies (DLB), the hallmark αS pathologies termed Lewy bodies and Lewy neurites, occur mostly in neurons. In comparison, in Multiple System Atrophy (MSA), αS inclusions, termed glial cytoplasmic inclusions, occur principally in oligodendrocytes although some neuronal αS inclusions, referred to as neuronal cytoplasmic inclusions, are also present (1, 2, 4, 5, 6). While αS phosphorylated at S129 is a common characteristic of these inclusions, various forms of αS carboxy (C)-truncated species invariantly accumulate to comprise 10 to 25% of αS found within pathological inclusions (7, 8, 9, 10, 11, 12) and are thought to be predominantly generated by lysosomal proteases (13, 14, 15, 16, 17, 18).

Supported by many lines of evidence, it is currently hypothesized that conformational templating of αS aggregation, akin to a prion-like mechanism, plays a significant role in the initiation of aggregation within cells and the intercellular progressive spread of αS pathology in the neuroaxis (2, 19, 20). Different non-mutually exclusive mechanisms can be involved in the prion-like intercellular transmission of αS inclusion pathology; however, this process can involve the cellular uptake *via* various endocytic pathways that converge on lysosomal-related organelles (15, 18, 21, 22, 23). In the experimental studies modeling the cellular uptake of αS, we have previously shown that exogenous human αS preformed fibrils (PFFs) can be extensively internalized and partially degraded by endosomal/lysosomal mechanisms in various cultured cells, resulting in specific cleaved forms of αS (18). Most of these cleavages appear to occur in the C-terminal region of αS, which is also the major region altered in αS that accumulates in human pathological inclusions (18, 21). In general, it has been posited that cleavage of the C-terminal regions of αS, which is unstructured and highly negatively charged, can promote and enhance its polymerization by significantly reducing intramolecular electrostatic repulsions that normally reduce its propensity to aggregate (21).

Using αS antibodies that we recently generated (6, 24), as well as the new antibodies generated herein, we identified the major αS cleavage products produced *via* the process of prion-type uptake of PFFs. We also demonstrate the enhanced aggregation properties of these C-truncated forms of αS as a likely mechanism that can promote inclusion formation. Furthermore, we demonstrated their presence in post-mortem human brain tissue from individuals with synucleinopathies and in animal models of prion-type induced αS pathology.

## Results

**Figure 1:**
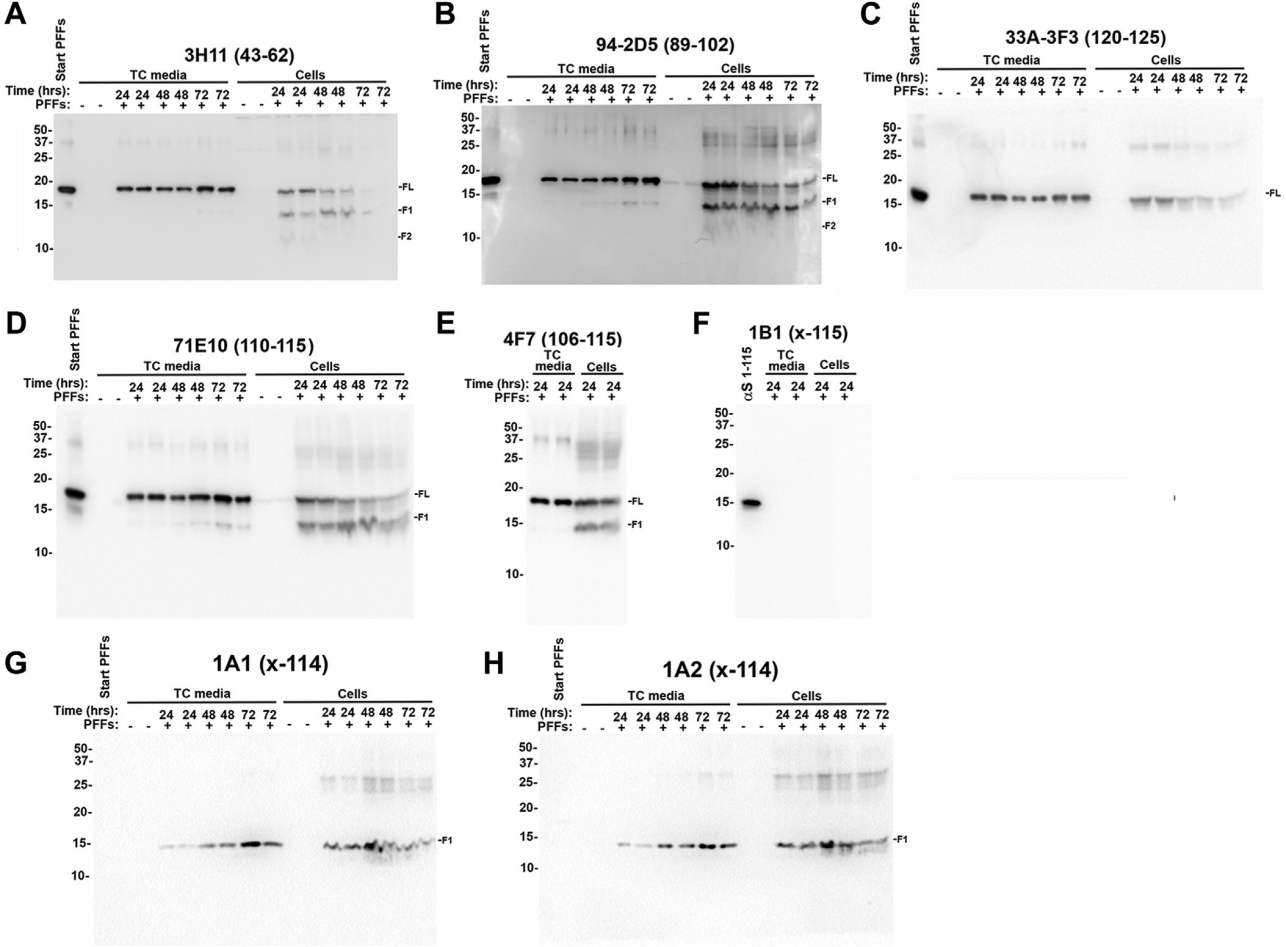
**Processing of exogenous human αS PFFs added to cultured HEK293T cells.** Cultured HEK293T cells were treated with 14 μg/ml FL αS PFFs for 24, 48, or 72 h as shown, in duplicate, as indicated above each lane. Untreated cultures (−) and start PFFs were included as controls. The TC media and total cell lysates were analyzed by immunoblotting with αS antibodies *A,* 3H11, *B,* 94-2D5, *C,* 33A-3F3, *D,* 71E10, *E,* 4F7, *F,* 1B1, *G,* 1A1 and *H,* 1A2. 30 ng of recombinant αS 1-115 was loaded as a positive control in *F*. The mobilities of molecular mass markers in kDa are indicated on the *left*. Similar results were observed in at least three independent experiments.

We previously showed that exogenous αS human PFFs can be internalized and degraded by endosomal/lysosomal mechanisms in various cultured cells (18). This process resulted in the formation of a major αS protein fragment with cleavage somewhere between residues 110 and 120 (18). Since we have generated additional antibodies with epitopes in the C-terminal region of αS, including antibodies specific for C-truncated forms of αS (6, 24), we wanted to further investigate this processing of αS (Fig. 1). Human αS PFFs were added to HEK293T cells for 24 to 72 h. The tissue culture (TC) media and cell lysates were analyzed with a series of αS antibodies. αS PFFs in the media that come into contact with cells can attach to cells and be internalized by endosomal/lysosomal mechanisms (18). Consistent with our previous findings, αS PFFs that remained in the media component were predominantly comprised of full-length (FL) αS, while αS PFFs that were associated with cells presented as FL αS, as well as a major processed fragment identified here as F1, and a minor processed fragment identified here as F2 detected with antibody 3H11, which targets an epitope (residues 43–62) in the middle of αS (Fig. 1*A*). These findings were further confirmed with αS antibody 94-2D5 with an epitope that includes residues 89-102 (Fig. 1*B*). Further characterization of the F1 fragment with a series of αS antibodies indicated that the F1 fragment did not react to 33A-3F3, an antibody targeting αS at residues 120-125 (Fig. 1*C*), but reacted with antibodies 71E10 and 4F7, which target epitopes at residues 110-115 and 106-115, respectively (Figs. 1, *D* and *E* and S1). However, the F1 fragment did not react with 1B1, an antibody specific for αS cleaved at residue 115 (24) (Fig. 1*F*).

**Figure 2:**
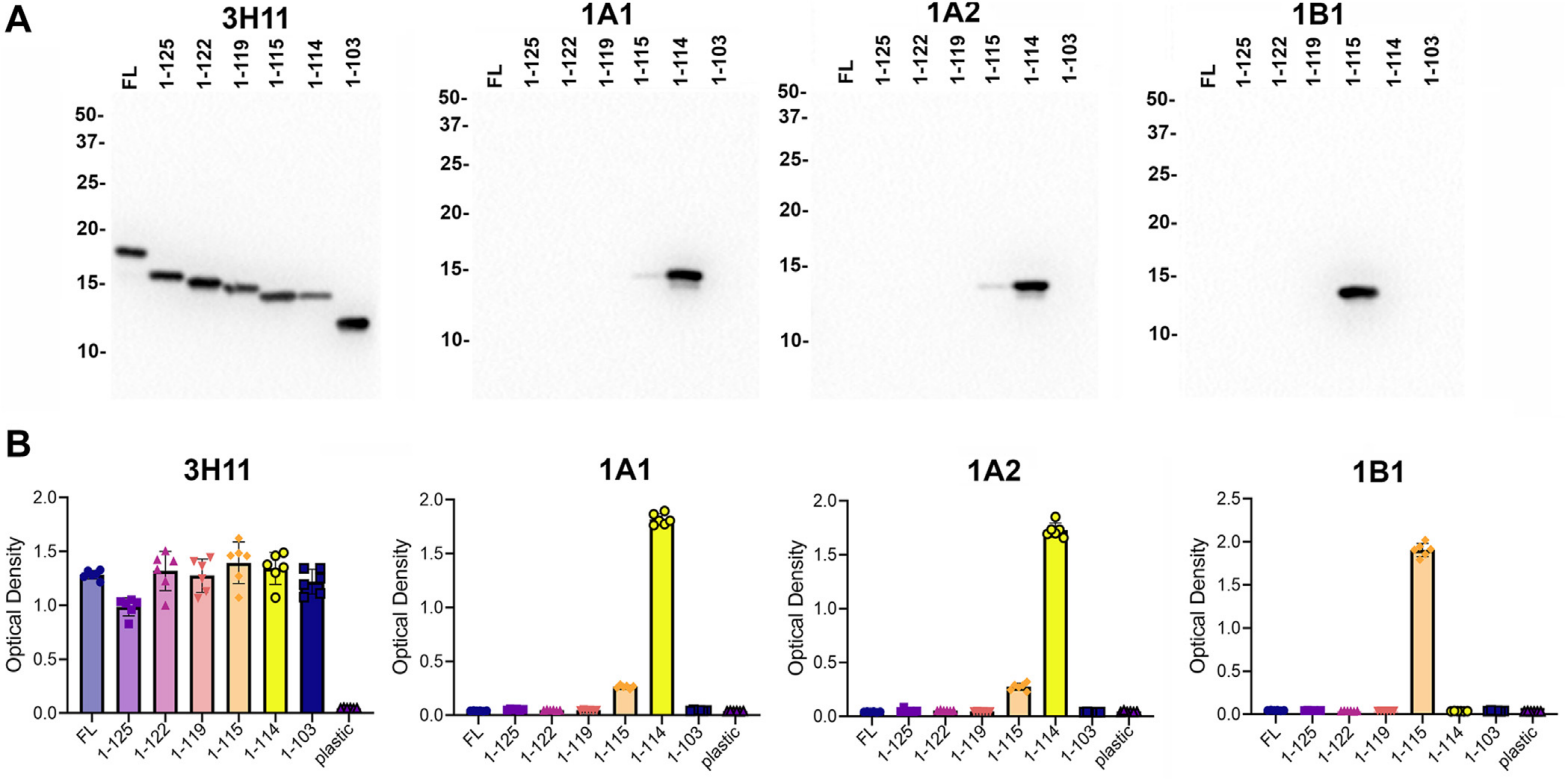
**Characterization of the specificity of new antibodies 1A1 and 1A2 to αS terminating at residue 114.***A*, immunoblotting analysis with antibodies 3H11 (43–62), 1A1, 1A2 and 1B1. As labeled above each lane 100 ng of recombinant FL or 1-125, 1-122, 1-119, 1-115, 1-114 and 1-103 αS were loaded in separate wells. The mobilities of molecular mass markers in kDa are indicated on the *left*. *B*, ELISA analysis with antibodies 3H11, 1A1, 1A2 and 1B1 was performed as described in “Experimental procedures” (n = 6). Error bars = SD. Similar results were observed in at least three independent experiments.

Lysosomal proteases that can be involved in the processing of αS have been reported to cleave αS after residues 114 and 115 (21, 25). To further assess the identity of the F1 product, we generated two novel antibodies 1A1 and 1A2 that can react with αS cleaved at residue 114. Both antibodies are IgG_1_ isotype. Immunoblotting and enzyme-linked immunosorbent assay (ELISA) analysis demonstrated that both antibodies were very selective for αS terminating at Glu114, with a slight reactivity for αS terminating at Asp115, but they did not react with human FL αS or αS truncated at residues 103, 119, 122 or 125 (Fig. 2). Antibody 1B1, which was previously characterized for its specificity for αS terminating at residue 115 (24), did not react with 1-114 αS (Fig. 2). The F1 fragment was shown to react with both 1A1 and 1A2 antibodies (Fig. 1, *G* and *H*) indicating that it is αS cleaved at residue 114.

**Figure 3:**
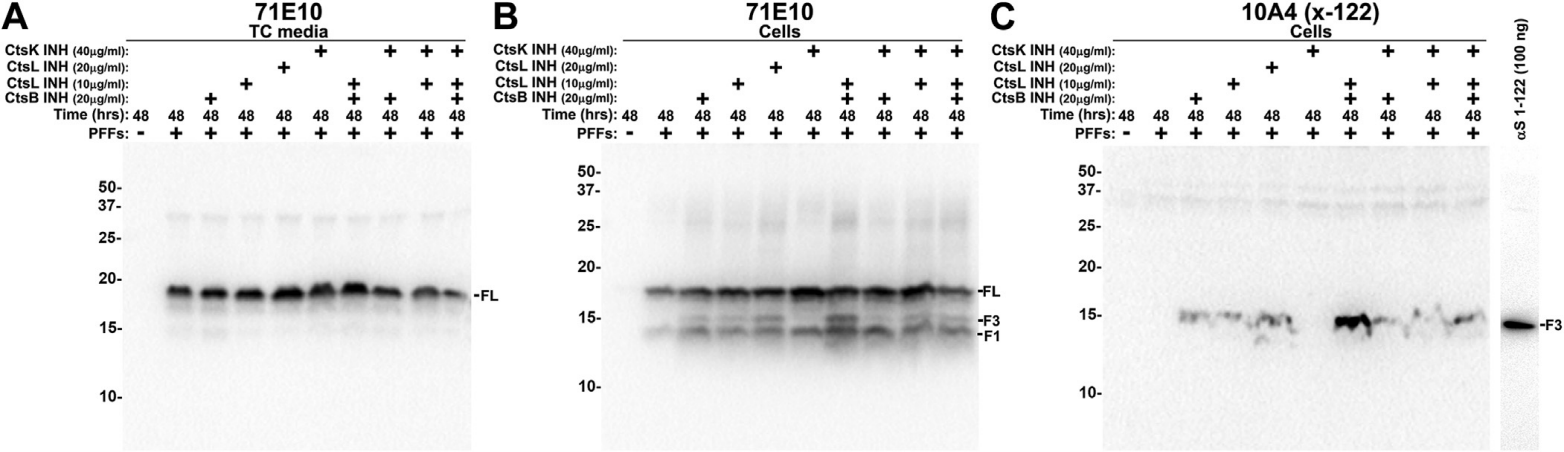
**Altered processing of internalized cellular PFFs by cathepsin inhibitors.** HEK293T cells were treated (+) or not treated (−) with PFFs with the addition of cathepsin (Cts) B, L or K inhibitors (INH), as indicated above each lane. *A*, TC media or cell lysates were analyzed by immunoblotting with antibodies *B,* 71E10 and *C,* 10A4, an antibody specific for αS carboxy truncated at residue 122. Recombinant human αS 1-122 (100 ng) was loaded in a separate lane as a positive control. The mobilities of molecular mass markers in kDa are indicated on the *left*. Similar results were observed in at least three independent experiments.

To investigate the effects of inhibiting lysosomal enzymes that might be involved in the generation of αS truncated at 114, cells that were incubated with PFFs were also treated with cathepsin B, L, or K inhibitors individually or combined (Fig. 3). These inhibitors did not have a major impact on the formation of the F1 fragment (x-114 αS), but cathepsin B and L inhibitors resulted in the accumulation of a new fragment termed F3 (Fig. 3, *B* and *C*) with cathepsin B and L inhibitors having a compounding effect. By immunoblotting analysis, the F3 protein fragment was not detected with antibodies specific for αS C-terminally truncated at residues 119 or 125 (24) (data not shown); however, probing with antibody 10A4, which is specific for αS truncated at residue 122 (24), revealed that the F3 fragment is αS cleaved at residue 122 (Fig. 3*C*).

**Figure 4:**
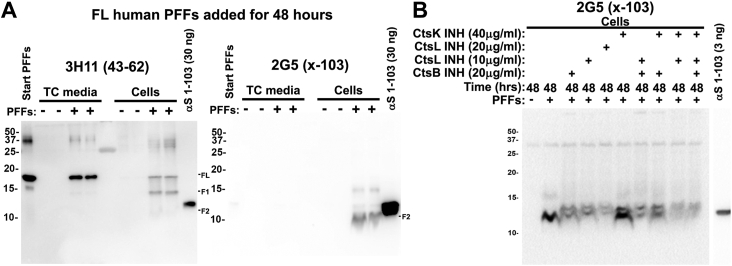
**Characterization of fragment F2 as αS cleaved at residue 103 with additional modifications.***A*, HEK293T cells were treated (+) or not treated (−) with PFFs and incubated for 48 h. TC media and cell lysates were analyzed by immunoblotting with αS antibody 3H11 (43–62) or 2G5, an antibody specific for αS cleaved at residue 103. Recombinant αS 1-103 was loaded as a positive control. The mobilities of molecular mass markers in kDa are indicated on the *left*. *B*, HEK293T cells were treated (+) or not treated (−) with PFFs with the addition of cathepsin (Cts) B, L or K inhibitors (INH), as indicated above each lane. Cell lysates were analyzed by immunoblotting with antibody 2G5. The mobilities of molecular mass markers in kDa are indicated on the *left*. Similar results were observed in at least three independent experiments.

The F2 fragment was reactive with antibodies 3H11 (residues 43–62) and 94-2D5 (residues 89–102) (Fig. 1, *A* and *B*) but was not detected with antibodies 71E10 (residues 110–115) or 4F7 (residues 106–115) (Fig. 1, *D* and *E*). To further investigate the identity of the F2 fragment, immunoblotting was performed with previously developed antibody 2G5, specific for αS truncated at residue 103 (24) (Fig. 4). Protein fragments detected with 2G5 (x-103) were approximately the molecular weight of the F2 fragment with a slight shift down, indicating that is also likely processed near the N-terminus (Fig. 4*A*). Treatment with cathepsin L and B inhibitors resulted in the formation of more pronounced immunoband doublet recognized by 2G5 with the upper band migrating slightly higher than the apparent molecular mass of recombinant αS 1-103 (Fig. 4*B*), indicating that other forms of post translational modifications are likely induced by these inhibitors. Treatment with cathepsin B, L, and K inhibitors did not prevent the formation of the F2 fragment (Fig. 4*B*).

**Figure 5:**
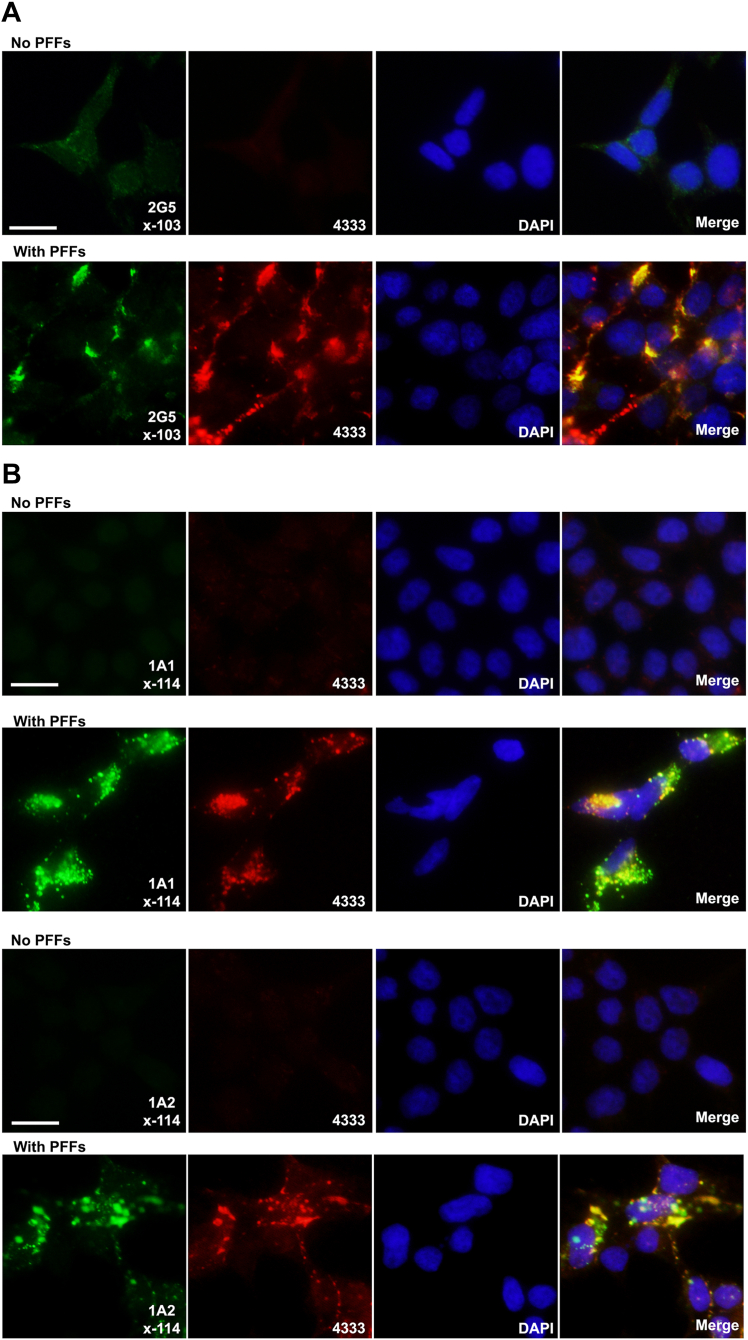
**Immunofluorescence analysis showing the intracellular accumulation of αS cleaved at residues 103 and 114 following PFF treatment.** HEK293T cells were untreated or treated with PFFs for 48 h as indicated above the panels and immunostained with mouse monoclonal antibody specific for αS cleaved at *A,* residue 103 (2G5) (shown in *green*) or *B,* residue 114 (1A1 and 1A2) (shown in *green*), and rabbit αS antibody 4333 (shown in *red*). Cells were also counterstained with DAPI to visualize the nuclei. Representative data from at least n = 3. Images were captured at 40× magnification and are representative of the 3 to 12 images per condition. Scale bar =10 μm.

Immunofluorescent analysis corroborated the accumulation of intracellular αS (4333 antibody staining) when cells were treated with αS PFFs and also revealed the intracellular formation of both x-103 αS (antibody 2G5) and x-114 αS (antibodies 1A1 and 1A2) (Fig. 5). The non-truncation specific αS antibody, 4333, showed a high prevalence of co-localization with all of these αS C-terminal truncation specific antibodies; 62% of cells showed 2G5+4333 positivity, 84% of cells showed 1A1+4333 positivity and 85% of cells showed 1A2+4333 positivity. This further indicates that this type of post-translational processing of αS is an integral property associated with intracellular uptake of αS PFFs.

**Figure 6:**
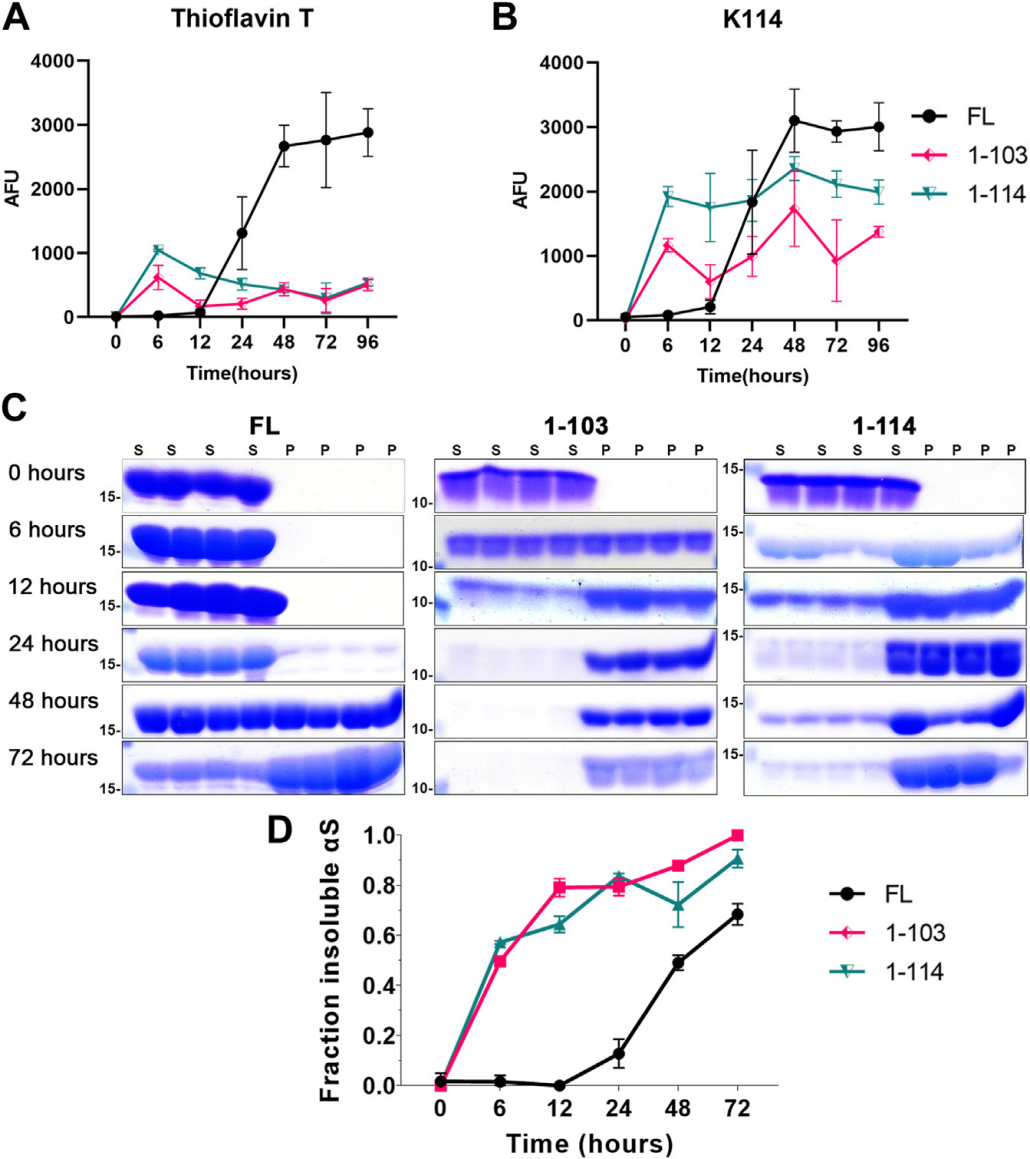
**1-103 and 1-114 αS aggregate faster than FL human αS but are less amyloidogenic.** Comparative temporal analysis of FL, 1-103 and 1-114 αS amyloid formation using *A,* Thioflavin T and *B*, K114 fluorometry. FL, 1-103, and 1-114 αS at 150 μM in PBS were incubated with agitation as described in “Experimental procedures” for the various time-points indicated (n = 4). *C*, protein aggregation was also assessed by sedimentation followed by Coomassie stained SDS-polyacrylamide gel analysis (n = 4). The mobilities of molecular mass markers in kDa are indicated on the *left*. *D*, densitometric analysis and statistical summary of the gels wherein which the insoluble fraction of each respective αS protein was determined as [αS in the pellet/(αS in the supernatant + αS in the pellet)]. Similar results were obtained in at least three independent experiments. Statistical summary provided in Table S1. Error bars = SD. AFU, arbitrary fluorescence units; P, pellet; S, supernatant.

Therefore, we sought to compare the aggregation kinetics and amyloid formation of 1-114 αS and 1-103 αS, respectively. These studies were performed using K114 and Thioflavin T fluorometry, as these assays can be used to monitor amyloid formation by interacting with different and independent amyloid binding sites (26, 27). Amyloid formation of monomeric FL, 1-103, and 1-114 αS with continuous shaking was compared over time (0–96 h) (Fig. 6, *A* and *B*). Amyloid formation increased rapidly within 6 h for αS fragments 1-103 and 1-114, at which time it was close to maximum for these two proteins. Comparatively, FL αS did not show initiation of amyloid formation until 12 h and did not reach close to maximal amyloid formation until 48 h (Fig. 6, *A* and *B*). However, FL αS reached substantially higher levels of amyloid formation compared to both 1-103 and 1-114 αS despite a slower rate of initiation (Fig. 6, *A* and *B*). αS polymerization was also assessed using a sedimentation assay which reiterated the findings that insoluble protein began to accumulate at 6 h for both truncated fragments, 1-103 and 1-114 αS, while substantial pellet formation did not begin until 24 h for FL αS (Fig. 6*C*). Interestingly, this analysis revealed an increase in pellet formation over time for all three proteins, with quantitative analysis revealing 1-103 αS having the greatest amount of insoluble αS levels by 72 h (Fig. 6*D*). Collectively, these findings indicate that while 1-103 and 1-114 αS fragments aggregate more rapidly compared to FL αS, the deletion of the respective C-terminal amino acid stretch for each protein altered the propensity to form typical amyloid structures.

**Figure 7:**
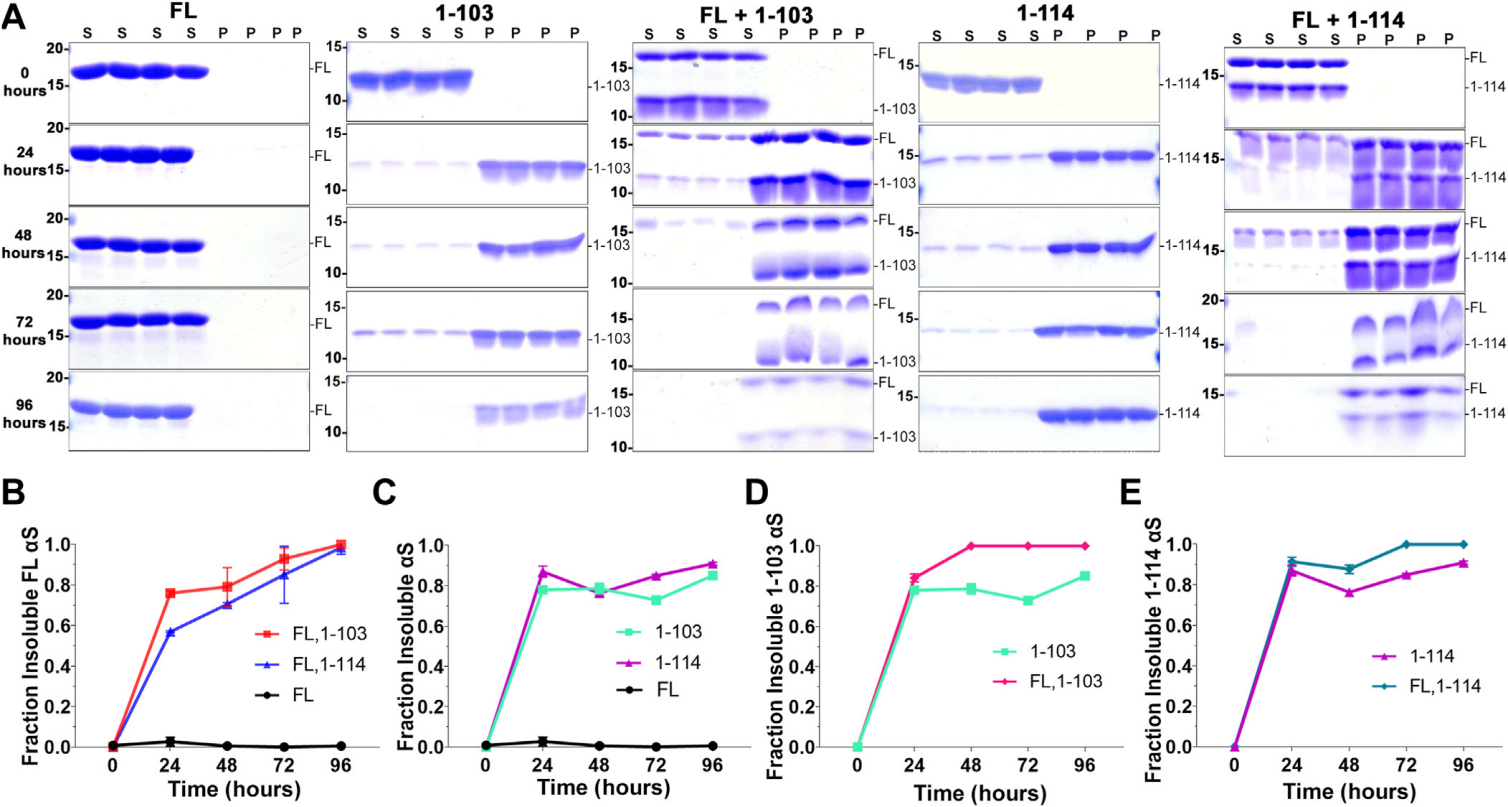
**1-103 and 1-114 αS promote the polymerization of FL human αS *in vitro*.***A*, FL, 1-103, and 1-114 αS alone or FL αS at a 1:1 M ratio with 1-103 or 1-114 αS (25 μM total) in PBS were incubated with agitation as described in “Experimental procedures” for the time-points indicated (n = 4). Supernatant (S) and pellet (P) fractions were analyzed by SDS-PAGE followed by Coomassie staining. The mobilities of molecular mass markers in kDa are indicated on the *left*. *B*–*E*, Fraction insoluble FL, 1-103, or 1-114 αS protein was determined for each specific protein as [αS in the pellet/(αS in the supernatant + αS in the pellet)]. Similar results were obtained in at least three independent experiments. Statistical summary provided in Table S2. Error bars = SD.

**Figure 8:**
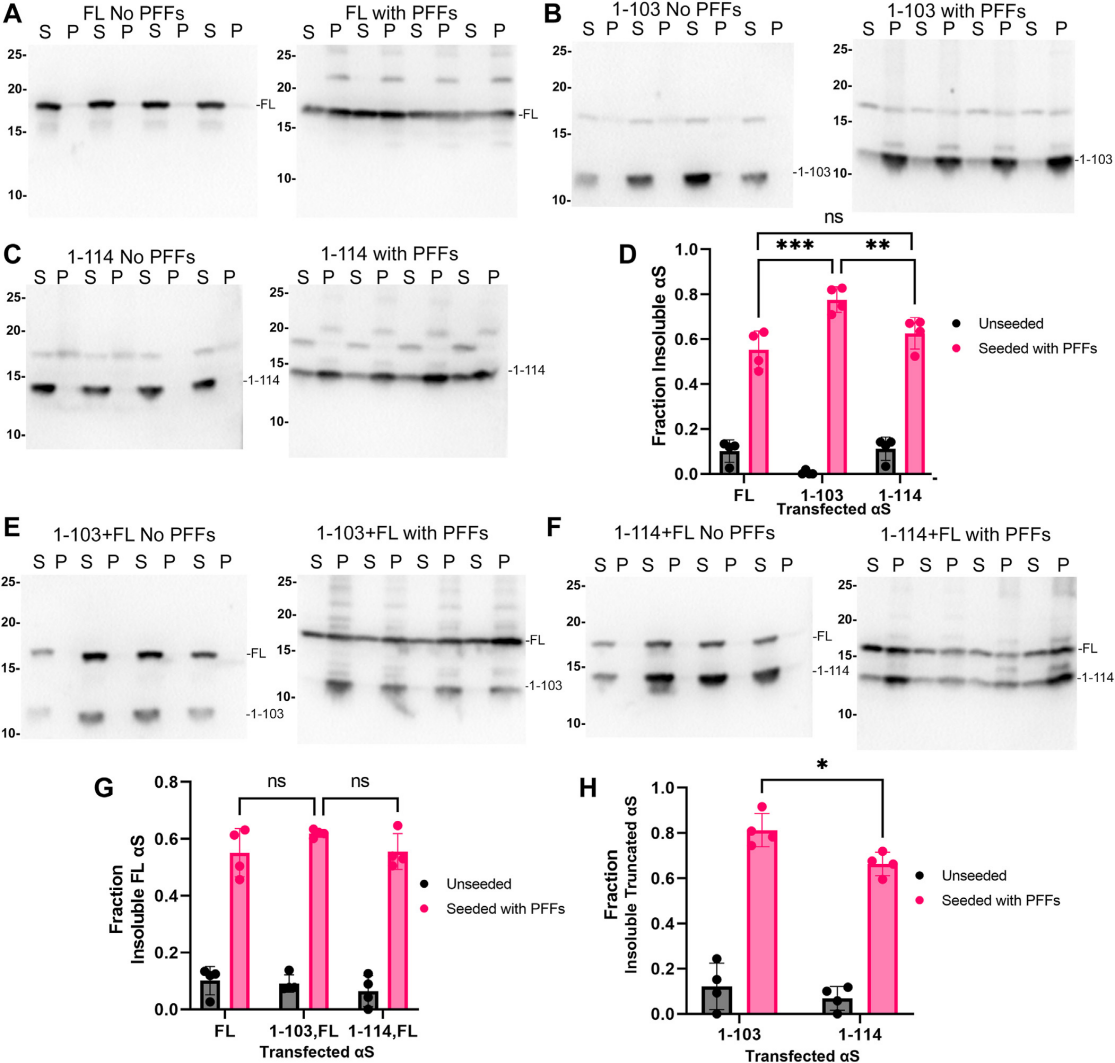
**1-103 and 1-114 αS expressed in HEK293T cells have a greater propensity to aggregate than FL αS in the presence of prion-type PFFs αS seeds.***A,* FL, *B*, 1-103, and *C*, 1-114 human αS were overexpressed in HEK293T cells using calcium phosphate transfection without or with the presence of exogenous PFFs comprised of human 21-140 αS as described in “Experimental procedures”. Cell lysates were fractionated into Triton X-100 soluble (S) and insoluble pellets (P) (n = 4). The cellular fractions were analyzed by immunoblotting with antibody SNL4, which does not react with 21-140 αS that was used to make PFFs for these studies. The mobilities of molecular mass markers in kDa are indicated on the *left*. *D*, densitometric analysis of blots in *A*–*C*; two-way ANOVA with Tukey’s test to determine the significance of the final extent of protein aggregation. The insoluble fraction of each respective αS protein was determined as [αS in the pellet/(αS in the supernatant + αS in the pellet)]. FL αS +PFFs *versus* 1-103 αS +PFFs (*p* = 0.0001), 1-103 αS +PFFs *versus* 1-114 αS +PFFs (*p* = 0.0053), FL αS +PFFs *versus* 1-114 αS +PFFs (*p* = 0.2016). Column factor (F = 583.1, *p* < 0.0001), rows defined as FL, 1-103, or 1-114 αS (F = 2.478, *p* = 0.1120), interaction accounting for 5.226% of total variance (F = 16.71, *p* < 0.0001). Error bars = SD. *E* and *F*, Western blot analysis of Triton-X-100 soluble (S) and insoluble pellet (P) fractions from cells co-transfected to overexpress FL with 1-103 or 1-114 human αS and untreated or treated with PFFs comprised of 21-140 αS fibrils. *G*, densitometric analysis of soluble *versus* insoluble fractions of FL αS from *A*, *E*, and *F*. The insoluble fraction of each respective αS protein was determined as [αS in the pellet/(αS in the supernatant + αS in the pellet)].With two-way ANOVA and Tukey’s test performed on data to determine the significance of the extent of FL αS aggregation. For quantification of FL fraction, FL+PFFs *versus* 1-103+FL+PFFs (*p* = 0.2093), 1-103+FL+PFFs *versus* 1-114+FL+PFFs (*p* = 0.2467). Column factor (F = 488.4, *p* < 0.0001) rows defined as FL, FL+1-103, or FL+1-114 αS (F = 1.429, *p* = 0.2656), interaction accounting for 0.4011% of total variance (F = 1.025, *p* = 0.3787). *H*, densitometric analysis of soluble *versus* insoluble fractions of 1-103 or 1-114 αS from the immunoblots in *E,* and *F*. The insoluble fraction of each respective αS protein was determined as [αS in the pellet/(αS in the supernatant + αS in the pellet)]. With two-way ANOVA and Šidák test performed on data to determine the significance of the extent of respective protein aggregation. For 1-103 and 1-114 αS fractions, 1-103+FL αS+PFFs *versus* 1-114+FL αS+PFFs (*p* = 0.0265). Column factor (*F* = 310.7, *p* < 0.0001) rows defined as 1-103 or 1-114 αS (*F* = 7.705, *p* = 0.0168), interaction accounting for 0.5278% of total variance (*F* = 1.753, *p* = 0.2102). Error bars = SD. ∗*p* ≤ 0.05; ∗∗*p* ≤ 0.01, ∗∗∗*p* ≤ 0.0001. Columns in each analysis defined as +PFFs or −PFFs. The mobilities of molecular mass markers in kDa are indicated on the *left*. Similar results were obtained in at least three independent experiments.

Since 1-103 and 1-114 αS are more prone to self-aggregation, we investigated whether the presence of these truncated forms of αS could enhance the polymerization of FL αS *in vitro*. These studies were performed at lower concentration (25 μM) of FL αS monomers and a 1:1 ratio of 1-103 and 1-114 αS monomers with FL αS. At this concentration, FL αS did not aggregate within the experimental timeframe (96 h) (Fig. 7, *A* and *B*), while 1-103 and 1-114 αS monomers still aggregated (Fig. 7, *A* and *C*) and when combined at a 1:1 M ratio, induced the polymerization of FL αS (Fig. 7, *A* and *B*). Additionally, combination of 1-103 or 1-114 αS with FL αS resulted in enhanced aggregation of the truncated αS relative to these truncated αS alone (Fig. 7, *D* and *E*). To examine how the presence of 1-103 or 1-114 αS affects seeding in cell culture, HEK293T cells were transfected with plasmids expressing 1-103, 1-114, FL αS, respectively or co-transfected with equal parts of 1-103 or 1-114 αS and FL αS expression vector. Cells were seeded with pre-formed 21-140 αS fibrils, allowing for the sole detection of the protein expressed in cells and not the exogenous PFFs with the antibody SNL4 (epitope amino acid 2–12). Expression of 1-114, 1-103, or FL αS by itself did not result in substantial αS aggregation, but treatment with exogenous PFFs induced αS aggregation across groups with significantly greater aggregation with 1-103 αS (Fig. 8, *A*–*D*). PFF treatment also induced the aggregation of FL and 1-103 αS or FL and 1-114 αS when cells were transfected to express both proteins, respectively (Fig. 8, *E*–*H*). Even in these co-expression studies, 1-103 αS was more prone to aggregate than FL or 1-114 αS however, 1-103 αS expression did not result in a significant increase in aggregation of FL compared to co-expression of 1-114 αS or FL alone (Fig. 8, *E*–*H*).

**Figure 9:**
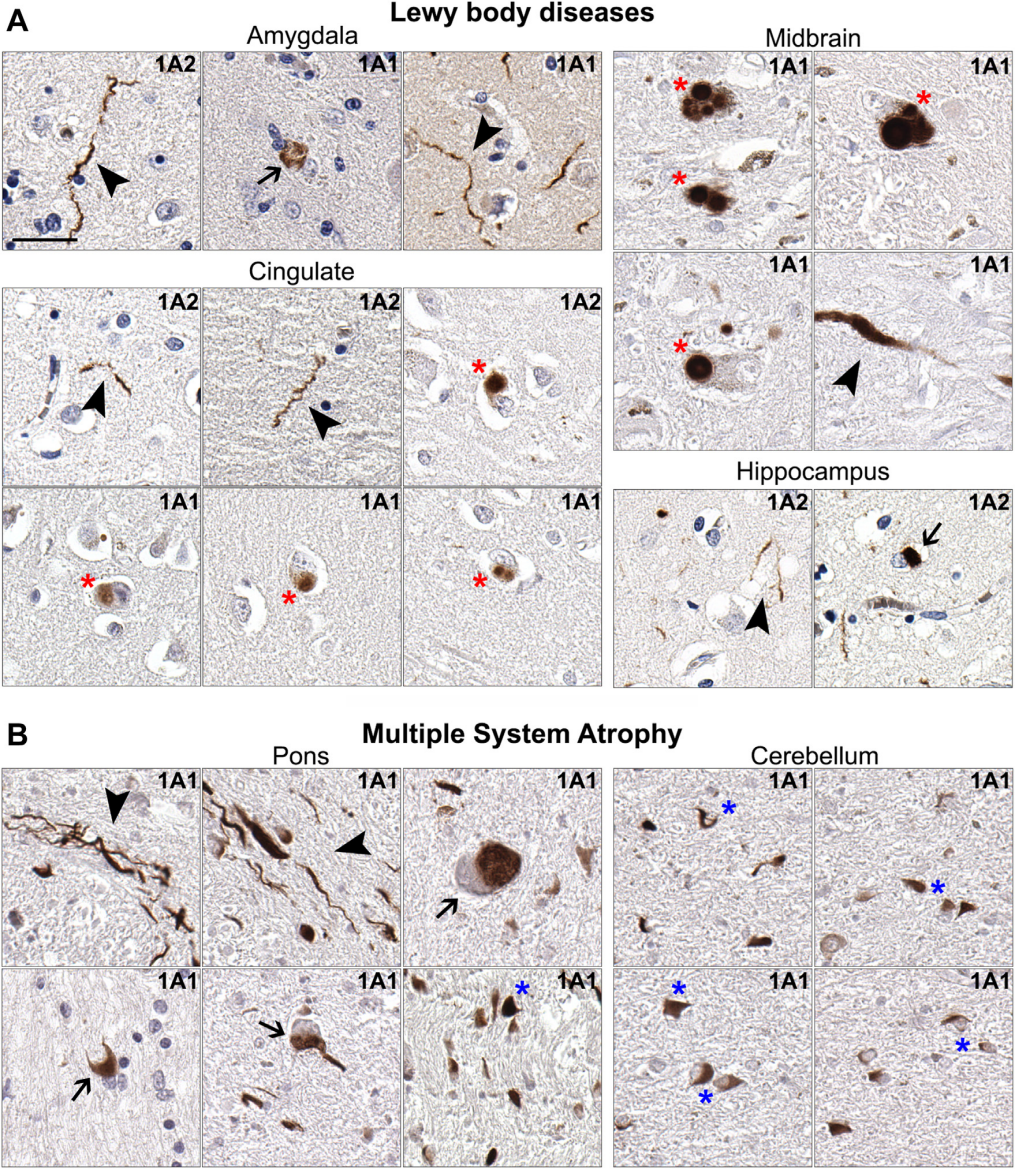
**Presence of x-114 αS within pathological inclusions of individuals with synucleinopathies.** Representative immunohistochemistry images labeled with antibodies 1A1 or 1A2 specific for x-114 αS as indicated in each panel from *A*, the amygdala, midbrain, cingulate or hippocampus of patients with LBD with neuronal inclusions (*arrows*), Lewy neurites (*arrow heads*) and various forms of Lewy bodies (*red asterisks*). *B*, representative immunohistochemistry images from the pons or cerebellum of patients with MSA with immunoreactive perikaryal (*arrows*) and neuritic (*arrow heads*) neuronal inclusions and glial cytoplasmic inclusions (*blue asterisks*). Sections were counterstained with hematoxylin. Scale bar = 30 μm for *A,* and *B*. See Table 1 for the cases that were used.

Immuno-reactivity of pathological inclusions in individuals with synucleinopathies was investigated with antibodies 1A1 and 1A2 (Table 1). In some patients with Lewy body disease (LBD), abundant x-114 αS immunoreactivity in Lewy bodies and Lewy neurites was observed, but these findings were not specific for any subtype of LBD (Fig. 9*A*; Table 1). Furthermore, in some patients with MSA, 1A1 and 1A2 immunoreactivity was observed in glial cytoplasmic inclusions in the cerebellum, as well as neuronal inclusions in the pons (Fig. 9*B*; Table 1). Pathology stained with antibodies 1A1 and 1A2 was observed in both formalin and ethanol fixed tissue.

**Table 1:** Summary of patient samples used for immunohistochemistry studies to investigate the presence of x-114 αS in pathological inclusion with antibodies 1A1 and 1A2

Pathological diagnosis	Total cases	Sex	Fixation of tissue used	Areas studied	Number of cases where x-114 staining was observed in pathological inclusion
LBD	8	7M/1F	3 formalin only/5 formalin and ethanol	Midbrain, cingulate, amygdala, hippocampus	5
MSA	7	4M/3F	4 formalin only/3 formalin and ethanol	Pons, cerebellum	3

LBD cases used include DLB, PD, PDD, and AD/ALB. Frontal cortex from a patient with Alzheimer’s disease was used as a negative control.

Abbreviations: AD/ALB, Alzheimer’s disease with amygdala predominant Lewy bodies; DLB, Dementia with Lewy bodies; LBD, Lewy body disease; MSA, Multiple System Atrophy; PD, Parkinson’s disease; PDD, Parkinson’s disease dementia.

**Table 2:** List of αS antibodies used with epitopes

Antibody name	Host	Epitope (residues)	References
3H11	Mouse	43–62	(56)
33A-3F3	Mouse	120–125	(57)
71E10	Mouse	110–115	(57) and Fig. S1
94-2D5	Mouse	89–102	(57)
4F7	Mouse	106–115	(6)
10A4	Mouse	122 carboxyl end specific	(24)
1B1	Mouse	115 carboxyl end specific	(24) and herein
2G5	Mouse	103 carboxyl end specific	(24)
1A1	Mouse	114 carboxyl end specific	herein
1A2	Mouse	114 carboxyl end specific	herein
4333	Rabbit	20–35	herein
SNL4	Rabbit	2–12	(58)

**Figure 10:**
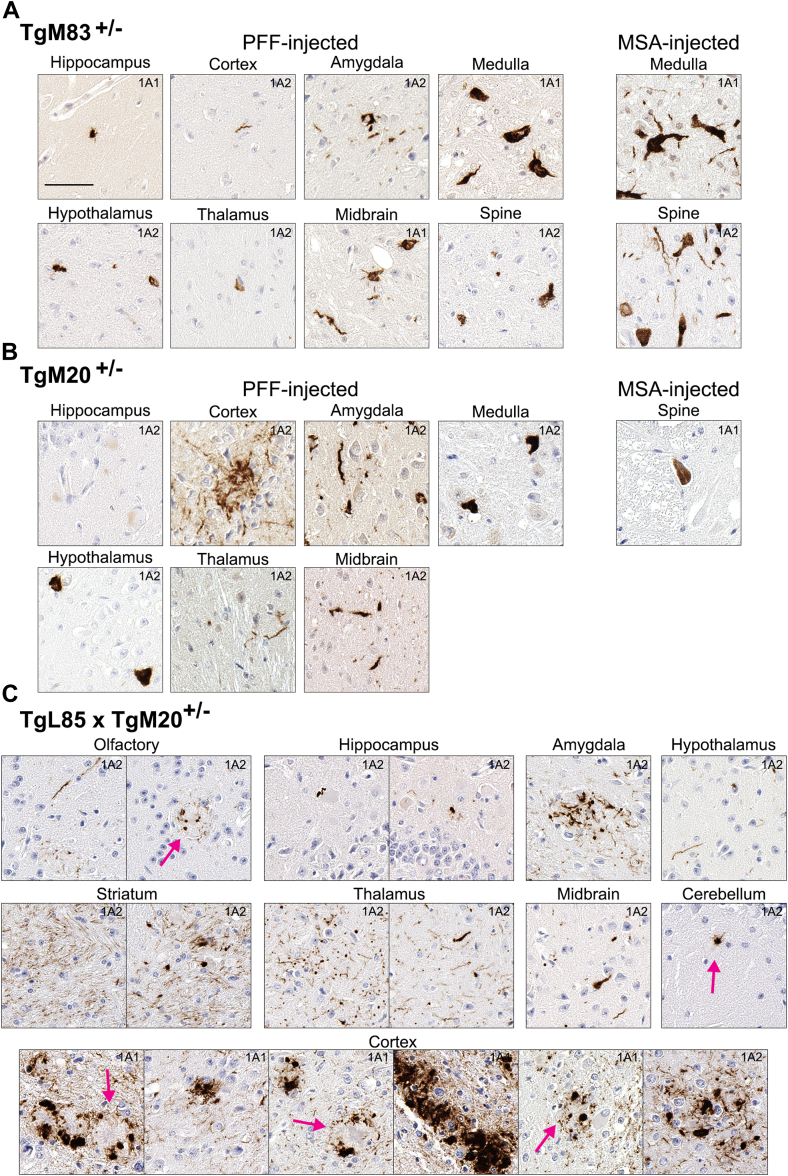
**Accumulation of αS truncated at residue 114 in mouse models of prion-type induced synucleinopathy.***A* and *B*, representative images of immunostaining with antibodies 1A1 or 1A2, which react with x-114 αS, in αS transgenic mice TgM83^+/−^ and TgM20^+/−^, wherein αS pathology was induced *via* the intracerebral injection of PFFs or MSA lysates in cohorts which were previously described (28). *C*, immunostaining with antibodies 1A1 or 1A2 in TgL85 × TgM20^+/−^ mice wherein αS inclusion pathology was induced by the intracerebral injection of PFFs (29). *Pink arrows* depict Aβ amyloid plaques. Sections were counterstained with hematoxylin. Scale bar = 50 μm for (*A*–*C*). Total number of samples analyzed = 16.

Immuno-reactivity of pathological inclusions in prion-like mouse models of synucleinopathies expressing human αS was investigated with antibodies 1A1 and 1A2 (Fig. 10, *A* and *B*). For transgenic mice expressing human A53T αS, termed TgM83^+/−^, we previously reported that CNS PFF-injection resulted in widespread αS inclusion pathology throughout the neuroaxis, with the highest levels occurring in the hippocampus, hypothalamus, periaqueductal gray (PAG), midbrain and medulla (28). Comparatively, x-114 αS reactive inclusion pathology was present in the CNS of these mice but not extensively abundant in the hippocampus, cortex, amygdala, hypothalamus, thalamus, and spine, while moderately present in the midbrain, PAG, and medulla (Fig. 10*A*).

For TgM83^+/−^ mice intracerebrally seeded with MSA lysates, we previously reported that αS pathology was prevalent in the hypothalamus, PAG, hindbrain and spinal cord while notably sparse in the cortex and hippocampus (28). Comparatively, x-114 αS pathology was only detected in the medulla and spine (Fig. 10*A*).

For transgenic mice expressing human WT αS, termed TgM20^+/−^, we reported that PFF hippocampal injection resulted in αS pathology which was abundant in the hippocampus, amygdala, ventral cortex, hypothalamus, PAG regions, medulla, midbrain and spine and mild in the thalamus (28). Comparatively, x-114 positive pathological inclusions were modest in the cortex and amygdala, sparse in the medulla, hypothalamus, thalamus, and midbrain, with rare and faint inclusions detected in the hippocampus (Fig. 10*B*). They were not detected in the spine of these mice. CNS αS pathology in TgM20^+/−^ mice seeded with MSA lysates showed a similar, albeit reduced regional trend as the PFF-injected mice (28); however, x-114 αS inclusions were only detected in the spine (Fig. 10*B*).

To further explore the inoculum and host-dependent patterns of x-114 pathology, we investigated a third prion-type, intracerebrally inoculated mouse model. In TgM20^+/−^ mice crossed with L85 mice, which accumulate Aβ plaques, we previously demonstrated that PFFs hippocampal injection induced CNS αS pathology, which was heavily potentiated by, and associated with, Aβ deposits (29). However, despite abundant hippocampal αS pathology, x-114 αS positivity was rare in the hippocampus (Fig. 10*C*). Mild x-114 αS positive pathology was observed in the olfactory bulb, midbrain and rarely cerebellum, and moderate x-114 αS positive pathology was observed in the amygdala, hypothalamus, striatum, and thalamus (Fig. 10*C*), which roughly correlated with the observed αS pathology (29). In the cortex, where αS pathology was ubiquitously abundant, x-114 αS pathology was mild in the dorsal regions and abundant in the ventral regions, especially, in the entorhinal and perirhinal cortex (Fig. 10*C*). Interestingly, x-114 αS positive pathology appeared to cluster around structurally conspicuous plaques (Fig. 10*C*, pink arrows). These plaque-contiguous clusters appeared as large dot-like neuropil inclusions encircling cored plaques (Fig. 10*C*, pink arrows).

## Discussion

C-terminal truncation of αS is an irreversible post-translational modification (PTM) that is highly associated with synucleinopathies and may play a role in the acceleration and toxicity of αS pathogenesis (21, 30, 31, 32). It has been proposed that the cell-specific and regional heterogeneity of αS found in MSA and LBD may be explained by the formation of disease specific αS with PTMs, such as C-terminal truncations, influencing the formation of unique αS pathologic conformers. These conformational differences in αS pathology are reflected in the variable ultrastructural and biochemical characteristics of MSA derived fibrils *versus* those of LBD (8, 33, 34, 35, 36). The relative abundance of C-terminally truncated αS found in post-mortem tissues from patients with LBD and MSA, and the increased aggregation propensity of C-terminally truncated αS *in vitro*, has drawn significant attention to this PTM as an early pathogenic step in synucleinopathies that may also confer the variability seen among synucleinopathies (11, 24, 37). Numerous studies have endeavored to characterize the physiological consequences of C-terminal truncations in neurodegenerative diseases whilst attempting to profile the PTMs most associated with respective synucleinopathies; however, these investigations have been hampered by the previously described conformational heterogeneity and a paucity of exploratory techniques (21).

### Generation of αS C-truncated at residues 114 and 103 from exogenous αS fibrils

Previously, while characterizing PFF seeding and subsequent endosomal/lysosomal processing in primary mouse neuronal-glial cell culture, we identified distinct αS fragments (termed F1 and F2 here), associated with prion-type uptake, lacking a portion of the C-terminus (18). Our lab has previously engineered novel monoclonal antibodies from various regions within the C-terminus of αS, as well as neoepitopes of αS, specifically C-terminally truncated αS (6, 24). In addition, herein we generated and characterized antibodies to x-114 αS. These tools were used to identify the F1 fragment as x-114 αS. Our current studies also discovered that the F2 fragment is formed by the cellular uptake of αS PFFs, and the cleaved product of αS is predominantly due to cleavage at residue 103. Furthermore, immunofluorescence staining showed that x-114 αS and x-103 αS co-localizes extensively with intracellular αS following cellular uptake of PFFs.

Interestingly, the application of several lysosomal enzyme inhibitors did not noticeably affect the formation of the F1 fragment (x-114 αS); however, treatment with cathepsin L and cathepsin B inhibitors produced the accumulation of a new αS fragment (F3) cleaved at residue 122. Multiple proteolytic enzymes including cathepsin L and cathepsin B (15, 21, 22) have been shown to cleave αS between residues 122/123 *in vitro*. The buildup of the x-122 fragment *via* treatment with cathepsin B and L inhibitors may be due to a compensatory shift in lysosomal proteolytic enzymes favoring its production. Alternatively, the processing of αS within lysosomes is likely sequential and the inhibition of cathepsin B and L may allow for the accumulation of x-122 αS as these enzymes may cleave αS at other sites (15, 22) while cleavage at 122 can still be carried by other lysosomal proteases. This altered accumulation is notable given that it was previously shown that 1-122 αS enhances aggregation of FL αS and amyloid formation *in vitro* (38). Co-expression of 1-122 αS with FL in cell culture also resulted in enhanced seeding culminating in larger fractions of insoluble FL (38). A shift in expression and activity of truncation producing proteases would be consistent with changes seen in the brains of LBD patients (39, 40). Similar to the F1 fragment, treatment with cathepsin L, B, and K inhibitors failed to prevent the formation of the F2 (x-103 αS) fragment that may be produced by asparagine endopeptidase (40). Treatment with cathepsin L and B inhibitors, however produced a second 2G5 positive band with a molecular weight more closely approximating that of recombinant 1-103 αS, indicating that the primary F2 fragment may have additional amino-terminal modifications resulting in the lower band of the doublet.

### Recombinant 1-114 and 1-103 αS show enhanced aggregation *in vitro*

While the seeding properties of 1-103 αS have been characterized by several studies with varying results, the characterization of x-114 has been limited (17, 32, 40, 41, 42). Previous studies have shown that various degrees of C-terminal truncation directly influence the conformational templating of FL αS, leading to greater amounts of insoluble αS in both *in vitro* sedimentation assays and cell seeding studies with 1-103 αS demonstrating unique seeding activity (38, 40). Our *in vitro* studies here show that, while 1-103 αS and 1-114 αS have enhanced aggregation kinetics compared to FL αS, they do not as robustly form amyloid structures to the extent of FL αS in isolation. These findings may signify that fibrils comprised solely of extensively C-terminally truncated αS interact differentially with K114 and Thioflavin T amyloid binding dyes. Given that the aggregation of αS is stochastic in nature, we sought to measure the effect of 1-103 and 1-114 αS on the aggregation of FL αS using low concentrations and conditions where FL αS by itself does not readily aggregate. Both 1-103 and 1-114 αS induced rapid aggregation of FL αS, indicating that their accumulation can potentiate the induction of αS aggregation. We similarly characterized the *in vitro* aggregation properties of 1-122 αS in previous studies, finding that it too aggregates rapidly and extensively compared to FL αS (38).

### Expressed 1-103 αS but not 1-114 αS shows enhanced aggregation in cultured cells

Cell culture studies have demonstrated that PFF-treated cells expressing 1-114 αS did not show significant propensity for aggregation compared to cells expressing FL αS, nor did it induce significant aggregation of FL αS when co-expressed with it within the short time course of the experiments herein. However, PFF treated cells expressing 1-103 αS did show enhanced aggregation compared to those expressing 1-114 αS or FL αS. Likewise, in PFF treated cells co-expressing 1-103 αS and FL αS, the fraction of 1-103 αS was almost entirely insoluble, whereas there was no difference in the insoluble fraction of FL αS**.** Taken together, these studies indicate that 1-103 αS and 1-114 αS have increased propensity to aggregate in some context, and influence the structure of αS aggregate formation. The effects of these truncated αS may be similar to an *in vitro* study which showed that 1-108 αS formed a strongly twisted fibril structure compared to that of FL, leading to more favorable templating of other truncated αS rather than FL (43), but this notion will require further investigation in future studies. While we did not seed with truncated αS fibrils in the current cellular experiments, the extent of 1-103 αS aggregation without significant increases in aggregation of co-expressed FL αS, may be due to the formation of intracellular 1-103 αS aggregates, which disrupt more extensive FL αS aggregation. Endogenous expression of C-truncated αS in this context may not completely replicate the conditions under which pathological aggregation occurs *in vivo*, and further animal studies may elucidate the role of these truncated αS in seeding αS pathology. Additionally, the proposed role of C-terminally truncated αS in αS pathology is not limited to the formation of intracellular inclusions. Indeed, several truncated αS species have been shown in both *in vitro* and *in vivo* models to mediate neuroinflammation, damage mitochondria, and increase vulnerability to oxidative stress (21, 44, 45).

### αS C-truncated at residue 114 in human neurodegenerative diseases

Previously, our lab revealed distinct patterns of C-truncated αS in synucleinopathies, using a panel of novel, truncation-specific αS antibodies (24). The antibodies were specific for αS truncated at residues 103, 115, 119, 122, 125 and 129. We found a constellation of region, cell, and disease-specific inclusions with these antibodies (24). In this study, we have expanded our arsenal of C-truncation-specific antibodies to include x-114 αS, revealing positive αS inclusions in the amygdala, midbrain, cingulate, and hippocampus of LBD cases as well as in the pons and cerebellum of MSA cases. Of note, x-114 αS positivity was only detected in a subset of patients; 5 out of 8 patients with LBD and 3 out of 7 patients with MSA. While our study aimed to analyze whether this truncation was present and therefore relevant to study in human disease, future studies should systematically explore the range of synucleinopathies, in order to elaborate upon the degree to which αS truncations occur, and investigate which forms are associated with greater pathogenicity in disease.

### αS C-truncated at residue 114 in transgenic mouse models of synucleinopathies

Overexpression and prion-like seeding mouse models of synucleinopathy have revealed an association between C-truncated αS and pathogenesis (11, 21, 46). We have previously reported several mouse models of prion-type induced synucleinopathies wherein which the intracerebral injections of PFFs or MSA lysates can induce the accumulation of αS inclusion pathology in the CNS of TgM20^+/−^and TgM83^+/−^ mice (28, 29, 47). Our previous findings revealed that both PFFs and MSA inoculum induced widespread αS inclusion pathology, but that the regional pattern and pathological burden was dependent upon the host genotype (28). Using our novel truncation-specific antibodies, we investigated whether x-114 αS was present in these cohorts, and whether the degree of x-114 αS specific pathology is influenced by mouse genotype or inoculum.

Interestingly, x-114 αS pathology considerably diverged from the previously observed pSer129 pathology in every model we studied. PFF injection into the hippocampus of TgM83^+/−^ mice resulted in some regions with high burdens of αS pathology, like the hippocampus and PAG, having relatively lower levels of x-114 αS pathology, whereas other regions with high αS pathology, like the medulla, showed moderate x-114 αS positivity. In this cohort, x-114 αS positive inclusion morphological subtypes included distorted nuclei, LB-like and ringed inclusions, dot-like neuropil and corkscrew neurites. Injection of MSA lysates into the hippocampus of TgM83^+/−^ mice resulted in a heavy burden of x-114 αS positive inclusions in the medulla and spine only. These mice presented with substantial αS pathology in the midbrain, PAG, and hypothalamus (28) that was not reactive for αS truncated at 114. X-114 αS positive inclusion morphological subtypes for TgM83^+/−^ MSA-injected mice included LB-like, flame-shaped, and globose inclusions, corkscrew, beaded and swollen neurites, axonal spheroids, dot-like neuropil and neuropil threads. Interestingly, while burden of αS pathology was comparable between PFF and MSA inoculum, in the medulla and spine, x-114 αS positivity did not follow this trend, as MSA-injected mice had a greater apparent burden in the medulla and spine compared to PFF-injected mice. One hypothesis to explain this finding could be that in TgM83^+/−^ mice, proteolytic events resulting in the generation of αS x-114 are more likely triggered by MSA lysate compared to PFFs.

For TgM20^+/−^ mice, PFF and MSA injection resulted in prolific αS pathology (28). However, in PFF injected mice, abundant x-114 positive αS inclusions were found only in the entorhinal and perirhinal cortex and in MSA injected mice x-114 αS positive inclusions were only in the spine. Furthermore, for PFF injected TgM20^+/−^ mice, x-114 αS positive inclusion morphological subtypes included LB-like inclusions, corkscrew and swollen neurites, dot-like neuropil and neuropil threads. For MSA injected TgM20^+/−^ mice flame-shaped inclusions were the major morphological subtype observed.

TgL85xTgM20^+/−^ mice injected with PFFs consistently displayed mild levels of x-114 αS positive inclusions in regions with moderate to high αS pathology, except for the hippocampus. Interestingly, among all mice included in this study, hippocampal x-114 αS laden inclusions were scarce relative to pSer129 positive inclusions (28, 29). Similar to PFF injected TgM20^+/−^ mice, TgL85xTgM20^+/−^ mice displayed high level of x-114 αS positivity in the entorhinal and perirhinal cortex, although alterations in morphology was detected amongst plaques. Other x-114 αS positive morphological subtypes included granulovacuolar bodies, LB-like, flame shaped, and globose inclusions, corkscrew, beaded, lightening and swollen neurites, axonal spheroids, dot-like neuropil and neuropil threads. One hypothesis to explain the abundance of x-114 αS positive pathology within the Aβ plaques of seeded TgL85xTgM20^+/−^ mice is that cellular processes altered by Aβ deposition may result in increased accumulation of x-114 αS.

It is plausible that the accumulation of C-truncated αS in mice injected with FL αS PFFs is the result of a proteolytic processing event in which the failed attempt to completely degrade aggregated αS results in cleaved αS. The detection of x-114 αS predominantly at regions distal to the site of seed injection relative to pS129 pathology implicates this PTM as an intermittent step in progressive prion-like transmission. However, confirmation of this temporal and spatial relationship of inclusion pathology requires further time-point studies. The full catalog of these αS fragments as they relate to pathological transmission is still under investigation, and it is probable that some fragments may potentiate prion-like fibrillization while others may result in a “dead-end” fibril, resistant to further elongation. Our data has demonstrated that x-114 αS positive pathological burden does not necessarily follow αS pathology in a linear fashion. Mitigating circumstances, such as primary amino acid sequence of endogenous αS, regional cellular propensity of αS cleavage, conformation of the initiating nucleation factor, or concurrent proteinopathies, may alter proteolytic processing events leading to the varied presence of x-114 αS. Furthermore, the human studies and the cohorts of mice used here display a limited temporal sequence in the relationship between initial αS prion-type seeding, transmission/spread of pathology, and the presence of x-114 αS. Future studies in mice will investigate the temporal presentation of x-114 αS, and other major αS C-terminal truncation products, relative to the initiation and spread of seeded αS inclusion pathology.

Although our studies implicate αS C-terminal processing in pathological αS transmission, additional investigations are greatly needed for the characterization of C-truncated αS fragments and their relationship to neurodegenerative diseases. Subsequent studies should elucidate the timeline of each fragment formation, and mitigating factors, which may promote certain truncations over others, such as region, cell type, co-proteinopathies or species of αS nucleation factor, and, importantly, relationship with onset and severity of disease.

## Experimental procedures

### Antibodies

See Table 2 for complete list of αS antibodies. 4333 is a rabbit antibody raised by immunization with a peptide corresponding to residues 20-35 in human αS with an added cysteine at the C-terminus (EKTKQGVAEAAGKTKEC) which was used for conjugation to maleimide-activated mariculture keyhole limpet hemocyanin (mcKLH) as a service provided by GenScript Biotech Corp.

### Generation of new monoclonal antibodies to αS cleaved at residue 114

Antibodies 1A1 and 1A2 are new mouse monoclonal antibodies generated by immunizing female BALB/c mice using a synthetic peptide (CEEGAPQEGILE) corresponding to amino acid residues 104-114 within the C-terminal region of human αS which was synthesized and purified by GenScript Biotech Corp. The peptide included an added cysteine residue at the amino terminus that allowed for conjugation to Imject maleimide-activated mcKLH (Thermo Scientific). The peptide-KLH conjugate was used to immunize female BALB/c mice (Jackson Laboratory) as previously described (48). All procedures were performed according to the NIH Guide for the Care and Use of Experimental Animals and were approved by the University of Florida Institutional Animal Care and Use Committee. Spleens from the mice were harvested, and the white bloods cells were fused with mouse myeloma cells (Sp2/O-Ag14; ATCC) as previously described (48). Hybridoma clones were selected using HAT supplement (Sigma Aldrich) and the surviving clones were initially screened for reactivity by ELISA using the respective peptide used for immunization, and further characterized using FL αS and a series of C-truncated αS proteins as detailed above. Antibody isotypes were determined using a mouse monoclonal isotyping kit (Millipore Sigma).

### Protease inhibitors

Cathepsin L inhibitor II (catalog number 219426) was purchased from Sigma-Aldrich, Inc. Cathepsin B inhibitor II (catalog number 21-938-5) and cathepsin K inhibitor II (catalog number 21-937-9) were purchased from Fisher Scientific.

### HEK293T Cell culture and calcium phosphate transfection

HEK293T cells were cultured in Dulbecco's modified Eagle medium high glucose (4.5 gm/L) supplemented with 10% fetal bovine serum, 100 U/ml penicillin, and 100 μg/ml streptomycin at 37 °C and 5% CO_2_. As described in the figures, some cells were treated with 14 μg/ml FL or 21-140 human αS PFFs. Cells were transfected using calcium phosphate transfection as previously described (38). For co-transfection, plasmids were at a 1:1 ratio keeping the total DNA used the same.

### Recombinant human αS proteins and preparation of human αS fibrils

Recombinant FL and C-terminally truncated human αS proteins were expressed in BL21 (DE3) *E. coli* (New England Biolabs Inc) using the pRK172 bacterial expression vector cloned with the human αS cDNA and the respective stop codon mutations for each C-terminal truncation as previously described (24, 38). 21-140 human αS was also expressed from the pRK172 plasmid with the cDNA from amino acids 21-140 with an added ATG initiation codon at the beginning. The new plasmid for the expression of 1-114 αS was created by introducing a stop codon at position 115 in the human αS cDNA *via* QuikChange site-directed mutagenesis and confirmed by Sanger sequencing. Recombinant FL (1–140), 21-140, and carboxyl truncated (1–103, 1–114, 1–115, 1–119, 1–122, and 1–125) human wild-type αS proteins were expressed from BL21 (DE3) *E. coli* (New England Biolabs Inc) and purified as described previously (24, 38, 49). Briefly, high salt (750 mM NaCl, 50 mM Tris, pH 7.5, 1 mM EDTA) and heat-resistant bacterial lysates were further purified utilizing size exclusion chromatography followed by Mono Q anion exchange chromatography for FL, 1-125, 1-122, 1-119 and 1-115 αS or Mono S cation exchange chromatography for 1-114 and 1-103 αS.

For generation of PFFs, αS proteins (5 mg/ml) were incubated in sterile PBS (Life Technologies) at 37 °C with continuous shaking at 1050 rpm (Thermomixer C, Eppendorf), and fibril formation was monitored by turbidity and K114 fluorometry (50). Fibrils were diluted to 2 mg/ml in sterile PBS and sonicated for 60 min in a water bath sonicator, which results in fragmentation into smaller fibrils of varying lengths to generate the αS PFFs added to the cultured cells (18, 50, 51).

For *in vitro* aggregation time course comparison studies, FL and C-truncated αS proteins were diluted to 150 μM in sterile PBS (Invitrogen) and assembled into amyloid fibrils with continuous shaking at 1050 rpm, at 37 °C, for 0, 6, 12, 24, 48, 72, and 96 h with four separate samples per protein at each time point. For coaggregation *in vitro* time course studies, proteins were diluted to a total of 25 μM, with FL and the respective C-truncated αS proteins inculcated alone or mixed at a 1:1 M ratio (FL:C-truncated αS).

Amyloid formation for each sample was assessed by K114 and Thioflavin T fluorometry as described previously (26, 27). K114 dye was diluted to 10 μM in 0.1 M glycine (pH 8.6) and fluorescence was measured at 550 nm emission with a filter cutoff of 530 nm and an excitation wavelength of 380 nm (26) using a Spectra Max Gemini spectrofluorometer and SoftMax Pro software (Molecular Devices). Thioflavin T dye was diluted to 100 μM in 0.1 M glycine (pH 8.6) and fluorescence was measured at 490 nm emission with a filter cutoff of 475 nm and excitation wavelength of 440 nm (27) using a Spectra Max Gemini spectrofluorometer and SoftMax Pro software. Additionally, insoluble polymer formation at 0, 6, 12, 24, 48, and 72 h was measured by centrifugation at 100,000*g* for 30 min at 4 °C. SDS-sample buffer was added to the separated pellets and supernatants to the same final volume and concentration (10 mM Tris, pH 6.8, 1 mM EDTA, 40 mM DTT, 0.005% Bromophenol Blue, 0.0025% Pyronin Yellow, 1% SDS, 10% sucrose). Supernatant and pellet samples were heated to 95 °C for 10 min, and equal volumes from each fraction were resolved by SDS-PAGE using 15% polyacrylamide gels. Gels were stained with Coomassie Brilliant Blue R-250 to visualize protein and destained in 10% isopropanol/10% acetic acid. Densitometric analysis of stained αS protein bands was conducted using ImageJ software to quantify the fraction of insoluble αsyn for each sample. Results from sedimentation studies were confirmed in three separate experiments producing similar results. Gel images were mounted using Adobe Photoshop and white leveled for uniformity in associated figures.

### Western blotting analysis

For analysis of TC media, 5X sample buffer was added to the media to a final 1X concentration (10 mM Tris, pH 6.8, 1 mM EDTA, 40 mM DTT, 0.005% Bromophenol Blue, 0.0025% Pyronin Yellow, 1% SDS, 10% sucrose) and samples were heated to 95 °C for 10 min. For analysis of total cell lysate, the cells were washed with PBS and directly lysed in sample buffer and heated to 95 °C for 10 min. Equal volumes of TC media or cellular lysates were resolved by SDS-PAGE on 15% polyacrylamide gels followed by electrophoretic transfer onto 0.2 μm pore size nitrocellulose membranes (Bio-Rad) in carbonate transfer buffer (10 mM NaHCO_3_, 3 mM Na_2_CO_3_, pH 9.9, 20% methanol) (52). Membranes were blocked in Tris buffered saline (TBS; 50 mM Tris, 150 mM NaCl, pH 7.5) containing 5% dry milk, and incubated overnight with primary antibodies in TBS/5% dry milk. Probing with primary antibodies was followed by goat anti-mouse conjugated horseradish peroxidase (Jackson Immuno Research labs). Protein bands were detected using Western Lightning Plus ECL reagents (PerkinElmer) followed by chemiluminescence imaging (PXi, Syngene). Quantified aggregation amounts used only experiments performed at the same time to reduce variability, although results were confirmed *via* three separate experimental replicates.

### HEK293T cells biochemical fractionation

HEK293T cells were washed once in PBS and lysed in 200 μl of detergent extraction buffer (25 mM Tris-HCl, pH 7.5, 150 mM NaCl, 1 mM EDTA, 1% Triton X-100, 20 mM NaF) supplemented with protease inhibitors (1 mM phenylmethylsulfonyl and 1 μg/ml each of pepstatin, leupeptin, N-tosyl-L-phenylalanyl chloromethyl ketone, N-tosyl-lysine chloromethyl ketone and soybean trypsin inhibitor). Detergent insoluble material was sedimented at 100,000*g* for 30 min at 4 °C and the supernatant was collected. To ensure complete removal of soluble material, centrifugation of the insoluble fraction was repeated before being suspended in the same volume of detergent extraction buffer as the supernatant. Sample buffer was added to both fractions and samples were heated to 95 °C for 10 min. The insoluble fractions were additionally probe sonicated for 1 min and boiled for an additional 10 min to ensure a homogenous solution prior to Western blot analysis.

### ELISA

The 96-well ELISA plates (Thermo Fisher) were coated with 100 ng recombinant αS proteins in 100 μl PBS per well. Wells were washed with PBS and blocked with TBS/5% skim milk powder. Primary antibodies were added to blocking solution and incubated at room temperature. After TBS washes, plates were incubated with horseradish peroxidase-conjugated anti-mouse antibody (Jackson Immuno Research Labs) in TBS/5% skim milk powder for 30 min. Plates were washed with TBS and 3,3′,5,5′-tetramethylbenzidine substrate (Thermo Fisher Scientific) was added to each well. The reactions were stopped by adding 0.5 M HCl and the optical density was measured at 450 nm with a plate reader.

### Immunofluorescence analysis

For immunofluorescence analyses, HEK293T cells cultured on coverslips were washed with PBS and fixed with 4% paraformaldehyde/PBS for 10 min. Following PBS washes, cells were blocked with 5% fetal bovine serum/PBS/0.1% Triton X-100 for 30 min. Cultures were incubated in primary antibodies followed by Alexa-fluor 488 and 594 conjugated secondary antibodies (Invitrogen). Nuclei were counterstained with 4′,6-diamidino-2-phenylindole (Invitrogen), and coverslips were mounted using Fluoromount-G (Southern Biotech). Experiments were replicated at least three times per condition with the analysis involving 3 to 12 representative images containing 29 to 247 cells per image captured at 40x magnification using an Olympus BX51 fluorescence microscope mounted with a DP71 digital camera (Olympus). Quantitative scoring was completed by two independent observers.

### Autopsy case material

Ethanol (70% ethanol/150 mM NaCl) and formalin-fixed brain samples of patients with DLB, Alzheimer’s disease (AD), Alzheimer’s disease with amygdala-predominant Lewy bodies (AD/ALB), MSA, Parkinson’s disease dementia and PD were provided by the University of Florida Neuromedicine Human Brain and Tissue Bank (UF HBTB) or the Center for Neurodegenerative Disease Research (CNDR) tissue bank at the University of Pennsylvania following institutional regulations (see Table 1). Postmortem diagnoses of LBD, MSA, AD neuropathological staging and other pathological diagnoses were made according to current guidelines and criteria proposed by the National Institute of Aging-Alzheimer’s Association (53), the Dementia with Lewy Bodies Consortium (54), and the Neuropathology Working Group on MSA (55).

### Mouse tissue of prion-type induced synucleinopathy

All animal experimental procedures were performed in accordance to the University of Florida Institutional Animal Care and Use Committee regulatory policies following approval. Mouse CNS tissue of prion-type induced models used here was previously described (28, 29).

### Immunohistochemistry

For immunohistochemistry, paraffin-embedded tissue on slides was rehydrated in xylene and a series of ethanol solutions (100%, 90%, and 70%). Tissue sections were placed in a steam bath for 60 min in a solution of modified citrate buffer (Target Retrieval Solution Citrate pH 6; Agilent), followed by extensive rinsing in running tap water. Endogenous peroxidases were quenched by submerging slides in TBS solution with 1.5% hydrogen peroxide and 0.005% Triton X-100. After washing with TBS, slides were blocked with 5% skim milk powder in TBS and incubated with primary antibody overnight at 4 °C. Slides were washed in TBS and blocked in 5% skim milk/TBS for 5 min, then incubated in a mixture of biotinylated secondary antibody (Vector Laboratories) in 5% milk/TBS (1:3000) and ImmPRESS polymer secondary antibody (Vector Laboratories) (1:10) for 1 h at room temperature. An avidin-biotin complex (ABC) system (Vectastain ABC Elite kit; Vector Laboratories) was used to enhance detection of the immunocomplexes, which were visualized using the chromogen 3,3′-diaminobenzidine (KPL). Tissue sections were counterstained with hematoxylin (Sigma Aldrich) followed by dehydration in ethanol solutions (70%, 90%, and 100%) and xylene before they were covered with Cytoseal (Thermo Fisher Scientific). Slides were digitally scanned and representative images were captured using an Aperio Slide Scanner AT2 instrument (40× magnification; Aperio Technologies Inc). Representative images were captured using Aperio ImageScope [v12.4.3.5008], and corrected for white balance, brightness and contrast levels using Adobe Photoshop CS3 (Adobe Systems).

## Data availability

The datasets and raw image files used and analyzed in the current study are available from the corresponding author upon reasonable request.

## Supporting information

This article contains supporting information.

## Conflict of interest

The authors declare that they have no conflicts of interest with the contents of this article.
