# Supplementary material for: Cellular processing of α-synuclein fibrils results in distinct physiological C-terminal truncations with a major cleavage site at residue Glu 114
Source: J Biol Chem. 2023 Jun 10;299(7):104912. doi: 10.1016/j.jbc.2023.104912 (PMC10404685; doi:10.1016/j.jbc.2023.104912)
Supplement: Supporting Tables S1 and S2 [file mmc1.pdf]

A. Thioflavin T				
**Time (hours)**	**Insoluble fraction comparison**	**95.00% CI of diff.**	**Summary**	**Adjusted P Value**
**6**	FL vs. 1-103	-991.3 to -200.1	*	0.0163
	FL vs. 1-114	-1055 to -981.4	****	<0.0001
	1-103 vs. 1-114	-818.1 to -27.10	*	0.042
**12**	FL vs. 1-103	-284.9 to 94.52	ns	0.2855
	FL vs. 1-114	-773.5 to -446.8	***	0.0003
	1-103 vs. 1-114	-716.2 to -313.7	***	0.0006
**24**	FL vs. 1-103	-61.37 to 2263	ns	0.0577
	FL vs. 1-114	-365.5 to 1952	ns	0.129
	1-103 vs. 1-114	-500.2 to -115.0	**	0.0065
**48**	FL vs. 1-103	1594 to 2871	***	0.0008
	FL vs. 1-114	1573 to 2908	**	0.0015
	1-103 vs. 1-114	-187.3 to 203.0	ns	0.9889
**72**	FL vs. 1-103	1014 to 3987	*	0.0106
	FL vs. 1-114	1008 to 3924	**	0.0095
	1-103 vs. 1-114	-506.6 to 437.3	ns	0.9715
**96**	FL vs. 1-103	1629 to 3123	**	0.0014
	FL vs. 1-114	1577 to 3109	**	0.0019
	1-103 vs. 1-114	-202.8 to 137.2	ns	0.8048

B. K114				
**Time (hours)**	**Insoluble fraction comparison**	**95.00% CI of diff.**	**Summary**	**Adjusted P Value**
**6**	FL vs. 1-103	-1279 to -879.4	***	0.0002
	FL vs. 1-114	-2154 to -1517	***	0.0002
	1-103 vs. 1-114	-1058 to -454.7	***	0.001
**12**	FL vs. 1-103	-900.6 to 114.3	ns	0.1047
	FL vs. 1-114	-2617 to -471.3	*	0.0176
	1-103 vs. 1-114	-2160 to -141.8	*	0.0321
**24**	FL vs. 1-103	-722.2 to 2409	ns	0.2424
	FL vs. 1-114	-1586 to 1531	ns	0.9978
	1-103 vs. 1-114	-1559 to -182.5	*	0.0191
**48**	FL vs. 1-103	195.1 to 2547	*	0.0274
	FL vs. 1-114	-205.5 to 1695	ns	0.1005
	1-103 vs. 1-114	-1771 to 518.0	ns	0.2262
**72**	FL vs. 1-103	742.4 to 3264	*	0.0125
	FL vs. 1-114	411.1 to 1222	**	0.0022
	1-103 vs. 1-114	-2426 to 53.37	ns	0.0568
**96**	FL vs. 1-103	877.5 to 2385	**	0.0049
	FL vs. 1-114	300.0 to 1724	*	0.0144
	1-103 vs. 1-114	-981.9 to -255.8	**	0.0078

C. αS Sedimentation				
**Time (hours)**	**Insoluble fraction comparison**	**95.00% CI of diff.**	**Summary**	**Adjusted P Value**
**6**	FL vs. 1-103	-0.5292 to -0.4310	****	<0.0001
	FL vs. 1-114	-0.6071 to -0.5051	****	<0.0001
	1-103 vs. 1-114	-0.09610 to -0.05582	***	0.0002
**12**	FL vs. 1-103	-0.8656 to -0.7151	****	<0.0001
	FL vs. 1-114	-0.7114 to -0.5766	****	<0.0001
	1-103 vs. 1-114	0.07194 to 0.2208	**	0.0023
**24**	FL vs. 1-103	-0.7733 to -0.5554	****	<0.0001
	FL vs. 1-114	-0.8229 to -0.5916	***	0.0002
	1-103 vs. 1-114	-0.1116 to 0.02573	ns	0.1712
**48**	FL vs. 1-103	-0.4468 to -0.3292	****	<0.0001
	FL vs. 1-114	-0.4070 to -0.05534	*	0.0215
	1-103 vs. 1-114	-0.02828 to 0.3420	ns	0.0767
**72**	FL vs. 1-103	-0.4041 to -0.2285	**	0.0013
	FL vs. 1-114	-0.3079 to -0.1375	***	0.0005
	1-103 vs. 1-114	0.01921 to 0.1680	*	0.0271

Supplementary Table 1. Statistical summary of Thioflavin T/K114 time-point study and *in vitro* aggregation rates

The ThT **(A)** and K114 **(B)** fluorescence reading for 1-103, 1-114, and FL αS at each time point **(Figure 6A and B)** and **(C)** the proportion of insoluble αS at each time point **(Figure 6D)** was compared using two-way ANOVA and Tukey's multiple-comparison test. NS, no significance; *, $p \leq 0.05$; **, $p \leq 0.01$; ***, $p \leq 0.001$; ****, $p \leq 0.0001$.

A. FL vs Mixed αS Sedimentation: Quantified FL αS

Time (hours)	Insoluble fraction comparison	95.00% CI of diff.	Summary	Adjusted P Value
24	(FL)FL vs. FL,1-103	-0.8082 to -0.6552	****	<0.0001
	(FL)FL vs. FL,1-114	-0.6191 to -0.4661	****	<0.0001
48	(FL)FL vs. FL,1-103	-0.8614 to -0.7084	****	<0.0001
	(FL)FL vs. FL,1-114	-0.7762 to -0.6232	****	<0.0001
72	(FL)FL vs. FL,1-103	-1.005 to -0.8515	****	<0.0001
	(FL)FL vs. FL,1-114	-0.9267 to -0.7737	****	<0.0001
96	(FL)FL vs. FL,1-103	-1.071 to -0.9181	****	<0.0001
	(FL)FL vs. FL,1-114	-1.055 to -0.9016	****	<0.0001

B. Truncated vs Mixed αS Sedimentation: Quantified Truncated αS

Time (hours)	Insoluble fraction comparison	95.00% CI of diff.	Summary	Adjusted P Value
24	(1-103)1-103 vs. FL,1-103	-0.08773 to -0.03534	****	<0.0001
	(1-114)1-114 vs. FL,1-114	-0.07131 to -0.01693	***	0.0005
48	(1-103)1-103 vs. FL,1-103	-0.2406 to -0.1882	****	<0.0001
	(1-114)1-114 vs. FL,1-114	-0.1406 to -0.08626	****	<0.0001
72	(1-103)1-103 vs. FL,1-103	-0.2981 to -0.2457	****	<0.0001
	(1-114)1-114 vs. FL,1-114	-0.1776 to -0.1232	****	<0.0001
96	(1-103)1-103 vs. FL,1-103	-0.1760 to -0.1236	****	<0.0001
	(1-114)1-114 vs. FL,1-114	-0.1178 to -0.06340	****	<0.0001

C. Single Sedimentation: Quantified αS

Time (hours)	Insoluble fraction comparison	95.00% CI of diff.	Summary	Adjusted P Value
24	FL vs. 1-103	-0.7939 to -0.7102	****	<0.0001
	FL vs.1-114	-0.8967 to -0.7880	****	<0.0001
	1-103 vs.1-114	-0.1439 to -0.03670	**	0.0095
48	FL vs. 1-103	-0.8318 to -0.7278	****	<0.0001
	FL vs.1-114	-0.7836 to -0.7312	****	<0.0001
	1-103 vs.1-114	-0.02772 to 0.07244	ns	0.3698
72	FL vs. 1-103	-0.7683 to -0.6879	****	<0.0001
	FL vs.1-114	-0.8636 to -0.8356	****	<0.0001
	1-103 vs.1-114	-0.1591 to -0.08385	***	0.0009
96	FL vs. 1-103	-0.8792 to -0.8104	****	<0.0001
	FL vs.1-114	-0.9190 to -0.8890	****	<0.0001
	1-103 vs.1-114	-0.09183 to -0.02657	**	0.006

Supplementary Table 2. Statistical summary of *in vitro* aggregation rates

The quantified proportion of insoluble αS at each time point **(Figure 7B-E)** was compared using two-way ANOVA and Šidák **(A and B)** or Tukey's **(C)** multiple-comparison test. NS, no significance; *, $p \leq 0.05$; **, $p \leq 0.01$; ***, $p \leq 0.001$; ****, $p \leq 0.0001$.
