# Supplementary material for: Cellular processing of α-synuclein fibrils results in distinct physiological C-terminal truncations with a major cleavage site at residue Glu 114
Source: J Biol Chem. 2023 Jun 10;299(7):104912. doi: 10.1016/j.jbc.2023.104912 (PMC10404685; doi:10.1016/j.jbc.2023.104912)
Supplement: Supporting Figure S1 [file mmc2.pdf]

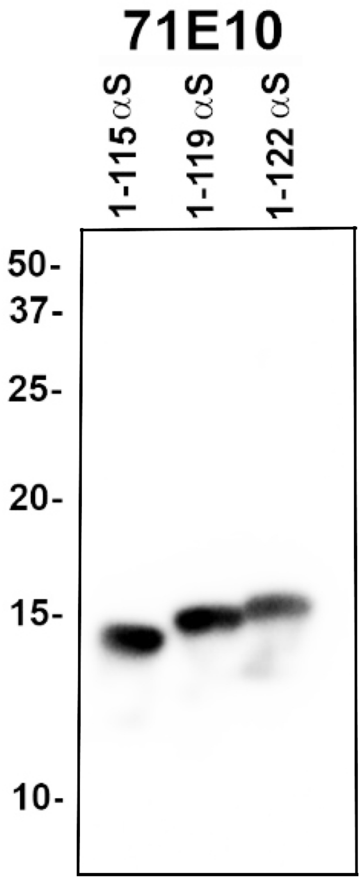

Supplemental Figure 1. Immunoblot analysis of antibody 71E10 to further refine the epitope. 30 ng of recombinant human 1-115, 1-119 and 1-122 αS was used for immunoblot analysis with antibody 71E10. The mobilities of molecular mass markers in kDa are indicated on the left.
